# AI-aided general clinical diagnoses verified by third-parties with dynamic uncertain causality graph extended to also include classification

**DOI:** 10.1007/s10462-021-10109-w

**Published:** 2022-01-29

**Authors:** Zhan Zhang, Yang Jiao, Mingxia Zhang, Bing Wei, Xiao Liu, Juan Zhao, Fengwei Tian, Jie Hu, Qin Zhang

**Affiliations:** 1 Institute of Nuclear and New Energy Technology, Tsinghua University, Beijing, China; 2 Department of General Internal Medicine, Peking Union Medical College Hospital, Chinese Academy of Medical Sciences & Peking Union Medical College, Beijing, China; 3 Department of ENT, Xuanwu Hospital of Capital Medical University, Beijing, China; 4 Department of Pulmonary and Critical Care Medicine, Xuanwu Hospital of Capital Medical University, Beijing, China; 5 Department of Gastroenterology, Beijing Hospital, Beijing, China; 6 Second Department of Liver Disease Centre, Beijing Youan Hospital, Capital Medical University, Beijing, China; 7 Chongqing Traditional Chinese Medicine Hospital, Chongqing, China; 8 Department of Medical Administration, Suining Central Hospital, Suining, Sichuan China; 9 Department of Computer Science and Technology, Tsinghua University, Beijing, China

**Keywords:** Clinical diagnosis, Classification, Generalization, Causality, Uncertainty, Probabilistic reasoning

## Abstract

Artificial intelligence (AI)-aided general clinical diagnosis is helpful to primary clinicians. Machine learning approaches have problems of generalization, interpretability, etc. Dynamic Uncertain Causality Graph (DUCG) based on uncertain casual knowledge provided by clinical experts does not have these problems. This paper extends DUCG to include the representation and inference algorithm for non-causal classification relationships. As a part of general clinical diagnoses, six knowledge bases corresponding to six chief complaints (arthralgia, dyspnea, cough and expectoration, epistaxis, fever with rash and abdominal pain) were constructed through constructing subgraphs relevant to a chief complaint separately and synthesizing them together as the knowledge base of the chief complaint. A subgraph represents variables and causalities related to a single disease that may cause the chief complaint, regardless of which hospital department the disease belongs to. Verified by two groups of third-party hospitals independently, total diagnostic precisions of the six knowledge bases ranged in 96.5–100%, in which the precision for every disease was no less than 80%.

## Introduction

AI-aided clinical diagnosis can help clinicians working at primary hospitals and clinics to avoid or reduce misdiagnoses and missing diagnoses. The ML models based on processed big data are well known, e.g., convolutional neural network (CNN), deep neural network (DNN), recurrent neural network (RNN) and Bayesian network (BN) (Fukushima and Neocognitron 1982; Lo et al. 1995; Russakovsky et al. 2015; Szegedy et al. 2015; Brosch et al. 2016; Shin et al. 2016; Duraisamy and Emperumal 2017; Bardou et al. 2018; Christodoulidis et al. 2017; Lin et al. 2018; Er et al. 2016; Ceccon et al. 2014), etc. However, most of them are applied to solve image and speech recognitions. AI-aided general clinical diagnosis is really needed in practice but is relatively rare. References (Wu et al. 2018) and (Liang et al. 2019) report two deep learning models that can perform general clinical diagnoses. However, it is not clear whether or not they have the same precisions when being applied in different application scenarios as being achieved in the testing dataset, which is called the generalization problem, although some comparisons between the models and clinicians have been made. The real world (primary level) applications are not qualified to judge the precisions, because of lacking the medical check measures, professional knowledge and experience. It is reasonable to doubt the generalization ability of the two models described in Wu et al. (2018) and (Liang et al. 2019), because the essence of deep learning is to establish a nonlinear mapping between the input (combinations of variable states including unknown states) and output (diseases) by adjusting the structure and parameters of the neural network. When the actual application scenario is different from the dataset in terms of sample space, which is common, the precision may drop, leading to the generalization problem.

In the general clinical diagnoses, there are at least 10,000 input variables. Each variable has at least 3 states: negative, positive and unknown. Thus, the number of state combinations of input variables are at least 3^10,000^ = 1.6 × 10^4771^, a huge number. The training and testing datasets cover only a small part of these state combinations, which is called the training and testing sample space (TTSS). The real application sample spaces (RASSs) are usually different from TTSS, while different application scenarios may have different RASSs. Thus, the mapping in TTSS may be different from that in RASSs. How the trained model based on TTSS can be applied in different RASSs needs to be verified. In fact, our experience is that the diagnostic precision drops significantly in real applications.

Moreover, how to ensure the model be able to diagnose the rare diseases is another problem, where the common diseases are relatively easy to be diagnosed even by primary clinicians and the rare diseases are really needed to be differentially diagnosed by the AI-aided models, which means that we need not only the high precision in total but also the high precision for each disease including rare diseases. Note that the common diseases are the majority in the training and testing datasets and the rare diseases may be marginalized in ML models, while the high precisions can still be achieved in the testing dataset due to the high proportion of common diseases. For the example of arthralgia shown in Table 4 in this paper, five common diseases (Gout, SLE, Osteoarthritis, RA and Trauma) have 95.8% case records in group 1, which implies that once the five diseases are correctly diagnosed, the total diagnostic precision will be 95.8%, even the diagnoses for the other 18 diseases are all incorrect. In practice, the correct diagnoses for the other 18 diseases are really needed.

Furthermore, because of the black box problem of deep learning models, the two models described in Wu et al. (2018) and (Liang et al. 2019) lack interpretability.

To solve these problems, the model based on the domain knowledge/causality is needed, because domain knowledge has invariance, which is essentially different from ML models basing on big data.

DUCG developed in recent years is such a model (Zhang 2012, 2015a, b; Zhang et al. 2014, 2018; Zhang and Geng 2015; Zhang and Zhang 2016; Zhang and Yao 2018) and has achieved promising application results for fault diagnoses of large, complex industrial systems (Zhang and Yao 2018; Zhang et al. 2018; Dong et al. 2014a, 2018; Qu et al. 2015; Zhao et al. 2014; Geng and Zhang 2014) and general clinical diagnoses (Dong et al. 2014b; Hao et al. 2017; Fan et al. 2018; Jiao et al. 2020; Ning et al. 2020; Zhang et al. 2021).

**Fig. 1:**
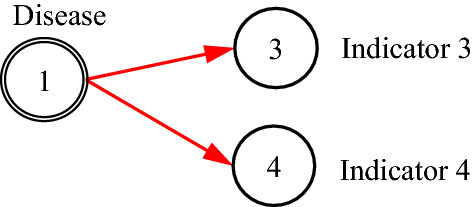
The case without *C*-type variable

**Fig. 2:**
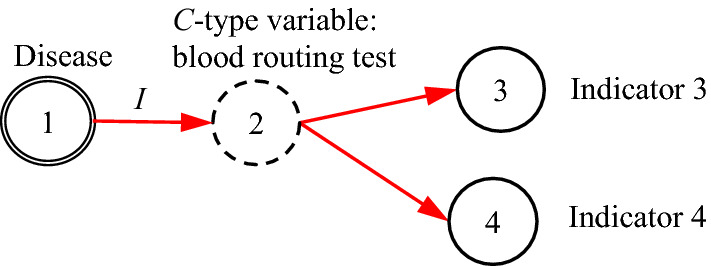
The case with *C*-type variable

It is noted that the existing DUCG model is entirely based on causalities. However, in many practical cases, some non-causal knowledge representations and associated probabilistic reasoning are needed. For example, when representing an uncertain causal relationship between a disease and a blood routing test, it is desirable to use the blood routing test as an inspection type variable, and to use the results of the test as its consequential variables. However, there is no direct causal relationship between the disease and the blood routing test itself, because the blood routing test is not the consequence of the disease. What actually exists is the uncertain causal relationship between the disease and the blood routing test results, i.e. the indicators. On the other hand, such causalities cannot be represented intuitively without the blood routing test variable, where the test is an action to find the consequences/indicators of diseases. In the hierarchical domain knowledge representation, the action is actually a classifier between the disease and the indicators. To solve this problem, the classification type (*C*-type) variable along with its unit matrix *I* drawn as its input directed arc is introduced as illustrated in Figs. 1 and 2.

It is proved in Sect. 3 that the DUCG without *C*-type variables is equivalent to that with them in the sense of inference. The former is resulted from the latter and is really used in the invisible DUCG inference, because the former is obviously easier to compute than the latter, while the latter remains as the visible knowledge base for better DUCG construction and interpretability.

Six DUCG knowledge bases including *C*-type variables for clinical diagnoses were constructed by clinical experts at Peking Union Medical College Hospital, Beijing Hospital, Xuanwu Hospital and Youan Hospital of Capital Medical University, Beijing, China. The diagnostic precisions were verified by two groups of third-party hospitals. Group 1 was Suining Central Hospital, Sichuan, China, which has a long history of more than 100 years. Group 2 was six hospitals officially organized as a whole by Chongqing Science and Technology Commission: West-South Hospital, Daping Hospital, The Second Affiliated Hospital of Chongqing Medical University, Chongqing Tumor Hospital, Chongqing Traditional Chinese Medicine Hospital (CTCMH) and Wanzhou Central Hospital, Chongqing, China. In which CTCMH was the leading unit. All hospitals are the Grade IIIA (the highest grade in China) hospitals and are located in southwest of China, far from Beijing where the knowledge bases were constructed. The verification results of the two groups are close to each other. Therefore, the generalization ability of DUCG were verified, which means that the DUCG-aided general clinical diagnoses can be applied in any application scenario without generalization problem that usually exists in ML models.

Section 2 introduces DUCG briefly. Section 3 presents the *C*-type variable methodology. Section 4 applies the *C*-type variable methodology to the diagnoses of six chief complaints. Two groups of third-party verifications were made. Section 5 summarizes this paper.

## Brief Introduction to DUCG

DUCG is a newly developed model that can explicitly and graphically represent causalities with uncertainties and perform probabilistic reasoning. In clinical diagnoses, it can easily represent various complex and uncertain causalities between diseases (root causes) and risk factors, symptoms, signs, image findings and laboratory results, etc., namely the observations or evidences. Conditional on the evidences collected for each patient, DUCG calculates the conditional probabilities of the found possible diseases, and thus performs intelligent diagnoses with clear casual and mathematical meanings (Zhang et al. 2021). To have the primary clinicians take responsibilities instead of DUCG, DUCG’s strong interpretability in knowledge bases, diagnostic results and computation process are very important.

**Fig. 3:**
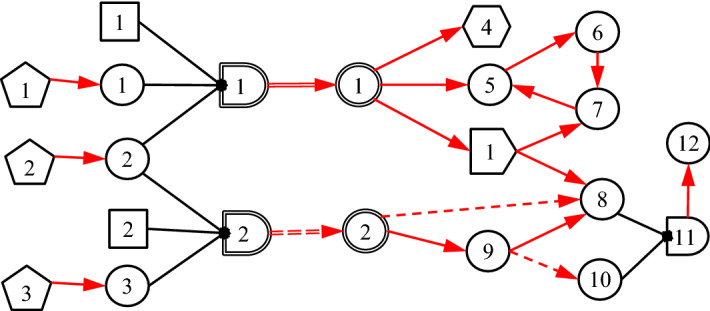
Illustrative DUCG

**Fig. 4:**
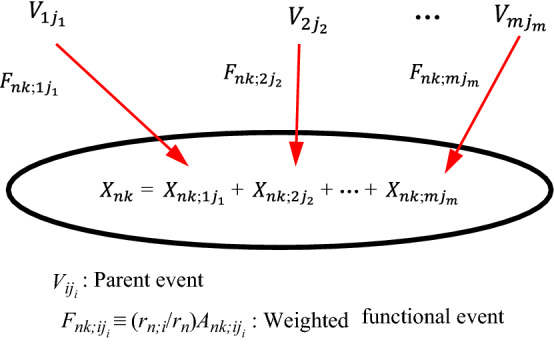
The basic idea of *M*-DUCG model (abbreviated as DUCG in this paper), in which *V* ∈ {*B*, *D*, *G*, *X*, *C*, *SX*, *BX*, *RG*}

DUCG is composed of two sub-models: single-valued DUCG (*S*-DUCG) and multivalued DUCG (*M*-DUCG). The so called single-valued means that only the causes of the true state of a child variable can be specified, while the false state is the complement of the true state. The so-called multivalued means that the causes of every state of a variable can be specified separately (Zhang 2012). In this paper, only *M*-DUCG is addressed and therefore is abbreviated as DUCG. Figure 3 is an illustrative DUCG. The symbols are described in Table 1. The basic idea of the DUCG model is shown in Fig. 4.

**Table 1:**
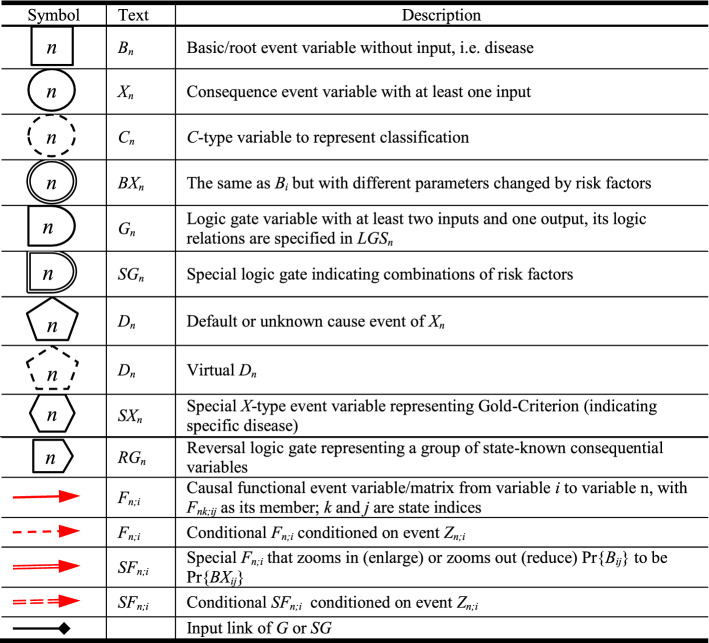
Graphical Symbols Used in DUCG

For simplicity, the subscript *j*_*i*_ in Fig. 3 is abbreviated as *j*. The rectangular node *B*_*n*_ is the basic or root cause event variable, without any input, and *B*_*nj*_ is state *j* of *B*_*n*_. The circular node *X*_*n*_ is the result event variable, *X*_*nj*_ is state *j* of *X*_*n*_, and *X*_*n*_ can be both the cause/input and the consequence/output of other nodes. The pentagonal node *D*_*n*_ is the default or unknown cause event of *X*_*n*_ or *X*_*nj*_, without any input, and its occurrence probability is defined as 1. The hexagonal node *SX*_*n*_ is a special *X*-type event variable, and *SX*_*nj*_ is state *j* of *SX*_*n*_. When *SX*_*nj*_ occurs, where *j* ≠ 0 and 0 indicates normal state, a particular disease or variable state must be true with a certain confidence *θ*, and therefore *SX*_*nj*_ is called gold-criterion in clinical diagnosis. The double-circle node *BX*_*n*_ is a *B*&*X*-type variable with both *B* and *X* properties. Its state division and definition are exactly the same as *B*_*n*_, and only the state probability distribution of *BX*_*n*_ may be different from *B*_*n*_ (affected by the associated risk factors). The logic gate variable *G*_*n*_ represents the various state combinations of the input variable and its input is connected with a directed arc . The double line logic gate *SG*_*n*_ represents various state combinations of the associated risk factors (such as age, gender, etc., represented as *X*-type variables), changing the state probability distribution of *B*_*n*_ as that of *BX*_*n*_. The output of *SG*_*n*_ is *BX*_*n*_, through a double-line directed arc  that zooms in or zooms out the state probabilities of *B*_*n*_ as that of *BX*_*n*_ according to the combinations of risk factors. The reversal logic gate *RG*_*n*_ drawn as  represents that the input of *RG*_*n*_ may cause some combinations of output. The single-line directed arc  represents the causality matrix *F*_*n;i*_ = (*r*_*n;i*_*/r*_*n*_)*A*_*n;i*_, where *A*_*nk;ij*_ is the element in the matrix *A*_*n;i*_, *A*_*nk;ij*_ is the virtual random event that the parent event *V*_*ij*_ (*V* ∈ {*B*, *X*, *D*, *G*, *BX*, *SX*}) causes the child event *X*_*nk*_ (including *SX*_*nk*_) directly. *r*_*n;i*_ > 0 is the strength of the causal relationship between *V*_*i*_ and *X*_*n*_, $r_{n} \equiv \sum\limits_{i} {r_{n;i} }$. The dashed directed arcs  or  is conditional  or  respectively, conditional on condition event *Z*_*n;i*_, where *n* indexes the child/output and *i* indexes the parent/input. When *Z*_*n;i*_ is true,  or  becomes  or  respectively; otherwise,  or  is eliminated.

In DUCG, the upper-case letter represents event or event variable and the corresponding lower-case letter represents the probability, i.e., *b*_*nj*_ = Pr{*B*_*nj*_}, *bx*_*nj*_ = Pr{*BX*_*nj*_}, *x*_*nj*_ = Pr{*X*_*nj*_}, *sx*_*nj*_ = Pr{*SX*_*nj*_}, *g*_*nj*_ = Pr{*G*_*nj*_}, *rg*_*nj*_ = Pr{*RG*_*nj*_}, *d*_*n*_ = Pr{*D*_*n*_}≡1, *z*_*n;i*_ = Pr{*Z*_*n;i*_}, *f*_*nk;ij*_ = Pr{*F*_*nk;ij*_} = (*r*_*n;i*_/*r*_*n*_)*a*_*nk;ij*_, *a*_*nk;ij*_ = Pr{*A*_*nk;ij*_}, *f*_*n;i*_ = Pr{*F*_*n;i*_}, *a*_*n;i*_ = Pr{*A*_*n;i*_}, etc. The indices before “;” are for the child and the indices after “;” are for the parent. The {*a*-, *b*-, *r*-}-type parameters are usually given by domain experts based on statistics or their experience. Note that the main formulas of DUCG are in the form of numerator divided by denominator (see (Zhang et al. 2021) for details). Therefore, only the relative values of parameters are sensitive, not the absolute values, which means that the parameters are easy to be given by clinical experts.

The variable index is inside the symbol without the letter of the variable type. The symbol shape represents the variable type. State index 0 denotes the normal/negative state, while the other states indicate abnormal/positive states. Moreover, *V*_*nj*_ ∈ {*X*_*nj*_, *SX*_*nj*_, *RG*_*nj*_}, *j* ≠ 0, is assigned with attention parameter *ε*_*nj*_ ≥ 1 that quantifies the attention of domain experts to explain the cause of *V*_*nj*_. If no cause can be found, a virtual *D*_*n*_ drawn as dashed pentagon will be assigned as the default cause of *V*_*nj*_ according to the DUCG simplification rule 10 listed in the Appendix of Zhang et al. (2021), and *a*_*nj;nD*_ between *V*_*nj*_ and the virtual *D*_*n*_ is defined as *a*_*nj;nD*_ = 1/*ε*_*nj*_, in which the index *D* indicates the invariable state of *D*_*n*_. In this case, *V*_*nj*_ is called the isolated evidence. Also, 0 < *θ*_*nj*_ ≤ 1 is assigned to *SX*_*nj*_ to quantify the confidence that the specific disease does exist given *SX*_*nj*_, where *j* ≠ 0. Ref. (Zhang et al. 2021) gives more details.

As shown in Fig. 4, the above events and probabilities satisfy Eqs. (1) and (2) respectively:

1 $$X_{nk} = \sum\limits_{i} {F_{nk;ij} V_{ij} } { = }\sum\limits_{i} {(r_{n;i} /r_{n} )} A_{nk;ij} V_{ij}$$

2 $$x_{nk} = \sum\limits_{i} {f_{nk;ij} v_{ij} } { = }\sum\limits_{i} {(r_{n;i} /r_{n} )} a_{nk;ij} v_{ij}$$

In which, *F*_*n;i*_≡(*r*_*n;i*_/*r*_*n*_)*A*_*n;i*_ and *f*_*n;i*_≡(*r*_*n;i*_/*r*_*n*_)*a*_*n;i*_. *F*_*nk;ij*_≡(*r*_*n;i*_/*r*_*n*_)*A*_*nk;ij*_, *f*_*nk;ij*_≡(*r*_*n;i*_/*r*_*n*_)*a*_*nk;ij*_ and *a*_*nk;ij*_ = Pr{*A*_*nk;ij*_}, where *F*_*nk;ij*_, *f*_*nk;ij*_, *A*_*nk;ij*_ and *a*_*nk;ij*_ are members of *F*_*n;i*_, *f*_*n;i*_, *A*_*n;i*_ and *a*_*n;i*_ respectively. In the case of only one input to *X*_*n*_, *F*_*n;i*_ = *A*_*n;i*_ and *f*_*n;i*_ = *a*_*n;i*_.

Equation (1) can be repeatedly applied until the expression becomes the sum-of-products composed of {*BX*-, *D*-, *A*-, *r*-}-type events and parameters, which is the event expanding process, and then the probability of the expression can be calculated by replacing the upper-case letters with the corresponding lower-case letters as illustrated in Eqs. (1) and (2). The state probability distribution of *BX*_*k*_ can be calculated from $bx_{km} = sa_{km;kj} b_{km}$, where *sa*_*km;kj*_ is the zoom factor transforming *b*_*km*_ to *bx*_*km*_ (see (7) in Zhang et al. (2021) for details). Then, *BX*-type variables can be treated as root causes/diseases.

The evidences can be written as $E = \bigcap\limits_{i} {X_{ij} }$. The diagnostic inference is to calculate the conditional probability Pr{*BX*_*kj*_|*E*} = Pr{*BX*_*kj*_*E*}/Pr{E}, *BX*_*kj*_ ∈ *S*_*H*_, *S*_*H*_ is the possible disease set conditional on *E*. We need to expand *E* as the sum-of-products composed of {*BX*-, *D*-, *A*-, *r*-}-type events and parameters. In which, logic computations such as absorption and exclusion and the *r*-type parameter calculation are applied.

In general, Eq. (3) is satisfied, in which “1” denotes complete set.

3 $$\sum\limits_{k} {A_{nk;ij} } = 1;\sum\limits_{j} {B_{ij} } = 1$$

Based on Eq. (3), we have the following theorem expressed as Eq. (4).

### Theorem 1

4 $$\sum\limits_{k} {X_{nk} = \sum\limits_{k} {\sum\limits_{i,j} {F_{nk;ij} V_{ij} } } } { = }\sum\limits_{i,j} {(r_{n;i} /r_{n} )\left( {\sum\limits_{k} {A_{nk;ij} } } \right)} V_{ij} = 1$$

Which means that the causality chains in DUCG are self-relied. Therefore, we do not need to specify all parameters in *a*_*n;i*_. For example, we may have Eq. (5).

5 $$a_{5;3} = \Pr \{ A_{5;3} \} { = }\left( {\begin{array}{*{20}c} {a_{5,0;3,0} } & {a_{5,0;3,1} } & {a_{5,0;3,2} } \\ {a_{5,1;3,0} } & {a_{5,1;3,1} } & {a_{5,1;3,2} } \\ {a_{5,2;3,0} } & {a_{5,2;3,1} } & {a_{5,2;3,2} } \\ \end{array} } \right) = \left( {\begin{array}{*{20}c} - & - & - \\ - & - & {0.{9}} \\ - & {0.{2}} & - \\ \end{array} } \right)$$

Which means that we can specify only the parameters in concern. In other words, for a variable whose state is normal (indexed by 0), we do not care about the causality and probability related to this state. What we are interested in is the causality between abnormal states. For example, a certain disease *B*_*ij*_ (*j* ≠ 0) causes a certain abnormal state *X*_*nk*_ (*k* ≠ 0), where *X*_*n*_ may represent a medical check result. We also do not care about the unconditional probability *b*_*i*0_ (i.e. without disease). That is to say, *b*_*i*0_, *a*_*n*0*;ij*_ and *a*_*nk;i*0_ in {*a*-, *b*-}-type matrices do not need to be given. Usually, we express *b*_*i*0_, *a*_*n*0*;ij*_ and *a*_*nk;i*0_ as “ − ” or blank, which is equivalent to null set in expanding *E*.

The DUCG diagnostic inference is to calculate the probability distribution of *BX*_*i*_ affected by risk factors observed for a patient and calculate Pr{*BX*_*kj*_|*E*} = Pr{*BX*_*kj*_*E*}/Pr{*E*}, *j* ≠ 0, in which *BX*_*kj*_ ∈ *S*_*H*_ is composed of the abnormal states of *BX*-type variables. *S*_*H*_ is the set of possible diseases conditional on *E*, and is found by the logical expanding and simplification of DUCG. The appendix in Zhang et al. (2021) lists the DUCG simplification rules. The detailed inference algorithm can be found in Zhang (2012)-(Zhang and Zhang 2016; Zhang et al. 2021).

## Introducing *C*-type Variables to Extend DUCG to Include Classification Relationship

### The basic idea

**Fig. 5:**
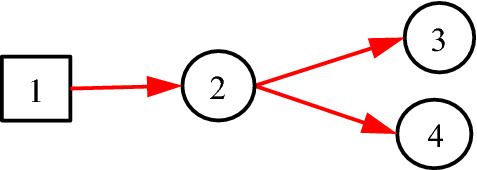
The taken-for-granted expression of pituitary prolactinoma

Consider Fig. 5, where *B*_1_ represents pituitary prolactin adenoma, *X*_2_ indicates whether thyroid function is normal, *X*_3_ indicates whether TSH (Thyroid Stimulating Hormone) is low, and *X*_4_ indicates whether FT3 (free triiodothyronine) is low.

**Fig. 6:**
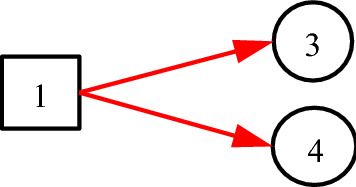
The actual **c**ausal relationship about pituitary prolactinoma

In Fig. 5, the hierarchy and relationships are clearly represented. It also embodies the medical knowledge of the disease, that is, pituitary prolactinoma (*B*_1_) may cause thyroid function abnormal (*X*_2_), and these abnormalities are manifested as TSH (*X*_3_) and FT3 (*X*_4_). However, problems are exposed when assigning values to the *a*-type matrices for each directed arc. Since *A*_2;1_ is a causal event matrix between pituitary prolactinoma *B*_1_ and thyroid function *X*_2_, *a*_2,1;1,1_ should be the probability of thyroid dysfunction caused by pituitary prolactinoma. Since *A*_3;2_ is a causal event matrix representing the causality from thyroid function *X*_2_ to TSH (*X*_3_), *a*_3,1;2,1_ should be the probability that thyroid dysfunction (*X*_2,1_) triggers low TSH (*X*_3,1_). Similarly, *a*_4,1;2,1_ should be the probability of thyroid dysfunction (*X*_2,1_) triggering low FT3 (*X*_4,1_). But this is obviously wrong, because the real causal relationship is: *X*_3,1_ and *X*_4,1_ are the causes of *X*_2,1_, not the opposite. At the same time, there is no direct causal relationship between *B*_1_ and *X*_2_. It is an indirect causal relationship with *X*_2_ through *X*_3_ and *X*_4_, and the direction is opposite. According to the expression in Fig. 5, the inference results of DUCG and the diagnosis results of clinical experts will be inconsistent, because the knowledge of the clinical experts is actually as shown in Fig. 6. In other words, Fig. 5 is incorrect. This example illustrates how easy the mistake may occur without classification variables.

To solve this problem, we introduce *C*-type variable along with *I* matrix as follows:

#### Definition 1

The state partition of the classification variable *C*_*n*_ drawn as  is identical to its parent variable *i*, *F*_*n;i*_ is fixed as a unit matrix $$I_{n;i} = \left( {\begin{array}{*{20}c} 1 & 0 & \cdots & 0 \\ 0 & 1 & \cdots & \vdots \\ \vdots & \vdots & \ddots & 0 \\ 0 & \cdots & 0 & 1 \\ \end{array} } \right)$$, and *F*_*m;n*_ is actually the causality between cause variable *i* and consequence variable *m*.

**Fig. 7:**
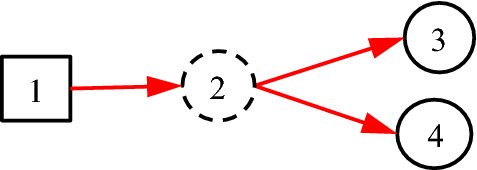
Using *C*-type variables to express classification relationships in DUCG

Equivalently, *f*_*n;i*_ = *I*_*n;i*_, because *f*_*n;i*_ = Pr{*F*_*n;i*_} = Pr{*I*_*n;i*_} = *I*_*n;i*_. Note that “1” in DUCG stands for both numerical one and complete set. With this definition, Fig. 6 can be better represented as Fig. 7.

In Fig. 7, according to Definition 1, *f*_2;1_ = *I*_2;1_, and *f*_3;2_ and *f*_4;2_ equal to *f*_3;1_ and *f*_4;1_ in Fig. 6 respectively.

#### Theorem 2

*In the sense of inference, the DUCG with C-type variable along with its corresponding I matrix is equivalent to the DUCG without C-type variables*.

Theorem 2 constitutes the inference algorithm of the DUCG with *C*-type variables, i.e. we can use the *C*-type variables along with *I* matrices to construct the DUCG with *C*-type variables, while the corresponding DUCG without *C*-type variables is really used in the DUCG inference. The latter is resulted from the former by (1) the elimination of *C*-type variables along with *I* directed arcs and (2) the connections between the cause and consequences of the *C*-type variable in the former. i.e., simplify Fig. 7 as Fig. 6. The inference equivalence is proved in follows:

#### Proof

First, we prove a simple case, i.e. Figures 6 and 7 are equivalent in inference. For this, we only need to prove that Pr{*B*_1_*X*_3_*X*_4_} in Fig. 6 and in Fig. 7 are equal. According to Fig. 6 and Eq. (1), we have.

6 $$\begin{gathered} \Pr \{ B_{1} X_{3} X_{4} \} = \Pr \{ B_{1} \left( {F_{3;1} B_{1} \cdot F_{4;1} B_{1} } \right) \} \\ = \Pr \{ (F_{3;1} *F_{4;1} )B_{1} \} \\ { = }(f_{3;1} *f_{4;1} )b_{1} \\ \end{gathered}$$

In which the operator “*” indicates to multiply the corresponding elements in the two matrices as defined in Corollary 15^1^ in Zhang et al. (2014). According to Fig. 7 and Eq. (1), we have

7 $$\begin{aligned} \Pr \{ B_{1} X_{3} X_{4} \} = & \Pr \{ B_{1} (F_{{3;2}} C_{2} \cdot F_{{4;2}} C_{2} )\} \\ = & \Pr \{ B_{1} (F_{{3;2}} *F_{{4;2}} )C_{2} \} \\ = & \Pr \{ B_{1} (F_{{3;2}} *F_{{4;2}} )I_{{2;1}} B_{1} \} \\ = & \Pr \{ (F_{{3;2}} *F_{{4;2}} )B_{1} \} \\ = & (f_{{3;2}} *f_{{4;2}} )b_{1} \\ = & (f_{{3;1}} *f_{{4;1}} )b_{1} \\ \end{aligned}$$

The last step in Eq. (7) is because *f*_3;2_ in Fig. 7 equals to *f*_3;1_ in Fig. 6, and *f*_4;2_ in Fig. 7 equals to *f*_4;1_ in Fig. 6. Thus, we have Eq. (7) equals to Eq. (6).

Obviously, the above proof can be applied in the case when the child variables of *B*_1_ in Fig. 6 and *C*_2_ in Fig. 7 are increased, which covers all cases of theorem 2. ■

According to Theorem 2, we can use Fig. 7 to express the medical hierarchical knowledge in the DUCG editor, automatically change Fig. 7 as Fig. 6 in the invisible inference, and perform the inference according to Fig. 6.

More details are addressed in follows.

### Single parent

**Fig. 8:**
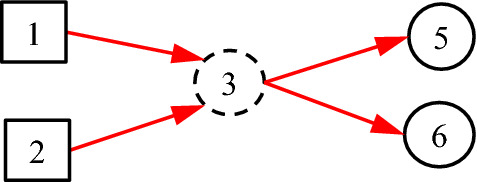
Incorrect expression for a *C*-type variable to have more than one parent variable

**Fig. 9:**
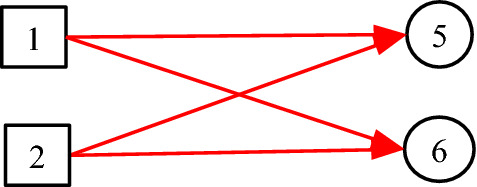
The real causalities behind Fig. 8

In Fig. 8, *C*_3_ has more than one parent, where the real causalities that we want to represent are as shown in Fig. 9. However, Fig. 8 may cause some trouble.

Suppose evidence *E* = *X*_5,1_*X*_6,2_, and *f*_5;3_ and *f*_6;3_ are given as follows:

$$f_{5;3} = \left( {\begin{array}{*{20}c} - & - \\ - & {f_{5,1;3,1} } \\ \end{array} } \right)$$, $$f_{6;3} = \left( {\begin{array}{*{20}c} - & - \\ - & {f_{6,1;3,1} } \\ \end{array} } \right)$$.

Based on Fig. 8, we have *f*_3;1_ = *I*_3;1_ and *f*_3;2_ = *I*_3;2_ as defined. According to Eq. (1), we have

8 $$\begin{aligned} \Pr \{ E\} = & \Pr \{ X_{{5,1}} X_{{6,2}} \} \\ = & \Pr \{ F_{{5,1;3,1}} C_{{3,1}} \cdot F_{{6,2;3,1}} C_{{3,1}} \} \\ = & \Pr \left\{ {\left( {F_{{5,1;3,1}} *F_{{6,2;3,1}} } \right)C_{{3,1}} } \right\} \\ = & \Pr \left\{ {\left( {F_{{5,1;3,1}} *F_{{6,2;3,1}} } \right)\left( {\frac{{r_{{3;1}} }}{{r_{3} }}I_{{3,1;1}} B_{1} + \frac{{r_{{3;2}} }}{{r_{3} }}I_{{3,1;2}} B_{2} } \right)} \right\} \\ = & \Pr \left\{ {\left( {F_{{5,1;3,1}} *F_{{6,2;3,1}} } \right)\left( {\frac{{r_{{3;1}} }}{{r_{3} }}B_{{1,1}} + \frac{{r_{{3;2}} }}{{r_{3} }}B_{{2,1}} } \right)} \right\} \\ = & \left( {f_{{5,1;3,1}} *f_{{6,2;3,1}} } \right)\left( {\frac{{r_{{3;1}} }}{{r_{3} }}b_{{1,1}} + \frac{{r_{{3;2}} }}{{r_{3} }}b_{{2,1}} } \right) \\ \end{aligned}$$

However, based on Fig. 9, we have

9 $$\begin{aligned} \Pr \{ E\} = & \Pr \left\{ {X_{{5,1}} X_{{6,2}} } \right\} \\ = & \Pr \left\{ {\left( {F_{{5,1;1}} B_{1} + F_{{5,1;2}} B_{2} } \right)\left( {F_{{6,1;1}} B_{1} + F_{{6,1;2}} B_{2} } \right)} \right\} \\ = & \Pr \left\{ \begin{gathered} F_{{5,1;1}} B_{1} F_{{6,1;1}} B_{1} + F_{{5,1;1}} B_{1} F_{{6,1;2}} B_{2} \hfill \\ + F_{{5,1;2}} B_{2} F_{{6,1;1}} B_{1} + F_{{5,1;2}} B_{2} F_{{6,1;2}} B_{2} \hfill \\ \end{gathered} \right\} \\ = & \Pr \left\{ \begin{gathered} \left( {F_{{5,1;1}} *F_{{6,1;1}} } \right)B_{1} + F_{{5,1;1}} B_{1} F_{{6,1;2}} B_{2} \hfill \\ + F_{{5,1;2}} B_{2} F_{{6,1;1}} B_{1} + \left( {F_{{5,1;2}} *F_{{6,1;2}} } \right)B_{2} \hfill \\ \end{gathered} \right\} \\ {\text{ = }} & \left( {f_{{5,1;1}} *f_{{6,1;1}} } \right)b_{1} + f_{{5,1;1}} b_{1} f_{{6,1;2}} b_{2} \\ & + f_{{5,1;2}} b_{2} f_{{6,1;1}} b_{1} + \left( {f_{{5,1;2}} *f_{{6,1;2}} } \right)b_{2} \\ \end{aligned}$$

Equation (9) is not equal to Eq. (8). To solve this problem, we have the following definition:

#### Definition 2

Each *C*-type variable can have only one parent variable, while different *C*-type variables may be the same in content.

**Fig. 10:**
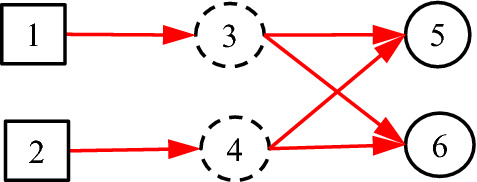
More than one *C*-type variables for more than one parent variable

Thus, Fig. 8 is changed as Fig. 10, in which *C*_3_ = *C*_4_. As defined, *f*_5;3_, *f*_5;4_, *f*_6;3_ and *f*_6;4_ in Fig. 10 equal to *f*_5;1_, *f*_5;2_, *f*_6;1_ and *f*_6;2_ in Fig. 9 respectively.

Based on Fig. 10, we have Eq. (10).

10 $$\begin{aligned} \Pr \{ E\} = & \Pr \left\{ {X_{{5,1}} X_{{6,2}} } \right\} \\ = & \Pr \left\{ {\left( {F_{{5,1;3}} C_{3} + F_{{5,1;4}} C_{4} } \right)\left( {F_{{6,1;3}} C_{3} + F_{{6,1;4}} C_{4} } \right)} \right\} \\ = & \Pr \left\{ \begin{gathered} F_{{5,1;3}} C_{3} F_{{6,1;3}} C_{3} + F_{{5,1;3}} C_{3} F_{{6,1;4}} C_{4} \hfill \\ + F_{{5,1;4}} C_{4} F_{{6,1;3}} C_{3} + F_{{5,1;4}} C_{4} F_{{6,1;4}} C_{4} \hfill \\ \end{gathered} \right\} \\ = & \Pr \left\{ \begin{gathered} \left( {F_{{5,1;3}} {\text{*}}F_{{6,1;3}} } \right)C_{3} + F_{{5,1;3}} C_{3} F_{{6,1;4}} C_{4} \hfill \\ + F_{{5,1;4}} C_{4} F_{{6,1;3}} C_{3} + \left( {F_{{5,1;4}} {\text{*}}F_{{6,1;4}} } \right)C_{4} \hfill \\ \end{gathered} \right\} \\ = & \Pr \left\{ \begin{gathered} \left( {F_{{5,1;3}} *F_{{6,1;3}} } \right)I_{{3;1}} B_{1} + F_{{5,1;3}} I_{{3;1}} B_{1} F_{{6,1;4}} I_{{4;2}} B_{2} \hfill \\ + F_{{5,1;4}} I_{{4;2}} B_{2} F_{{6,1;3}} I_{{3;1}} B_{1} + \left( {F_{{5,1;4}} *F_{{6,1;4}} } \right)I_{{4;2}} B_{2} \hfill \\ \end{gathered} \right\} \\ = & \Pr \left\{ \begin{gathered} \left( {F_{{5,1;3}} *F_{{6,1;3}} } \right)B_{1} + F_{{5,1;3}} B_{1} F_{{6,1;4}} B_{2} \hfill \\ + F_{{5,1;4}} B_{2} F_{{6,1;3}} B_{1} + \left( {F_{{5,1;4}} *F_{{6,1;4}} } \right)B_{2} \hfill \\ \end{gathered} \right\} \\ = & \left( {f_{{5,1;1}} *f_{{6,1;1}} } \right)b_{1} + f_{{5,1;1}} b_{1} f_{{6,1;2}} b_{2} \\ & + f_{{5,1;2}} b_{2} f_{{6,1;1}} b_{1} + \left( {f_{{5,1;2}} *f_{{6,1;2}} } \right)b_{2} \\ = & \left( {f_{{5,1;1}} *f_{{6,1;1}} } \right)b_{1} + f_{{5,1;1}} b_{1} f_{{6,1;2}} b_{2} \\ & + f_{{5,1;2}} b_{2} f_{{6,1;1}} b_{1} + \left( {f_{{5,1;2}} *f_{{6,1;2}} } \right)b_{2} \\ \end{aligned}$$

It is seen that Eq. (10) equals to Eq. (9), which means that Fig. 10 is equivalent to Fig. 9 in the sense of inference. In conclusion, Fig. 8 is not allowed and Fig. 10 should be used.

**Fig. 11:**
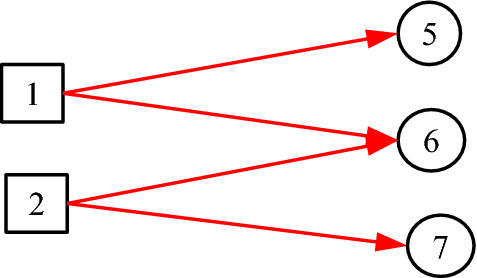
The causalities between causes and consequences/indicators

**Fig. 12:**
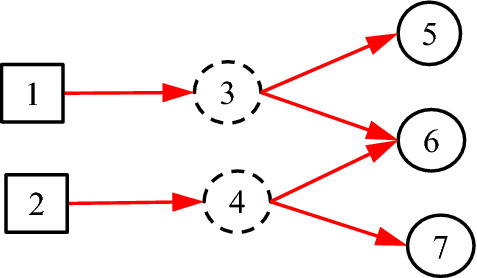
The corresponding DUCG with *C*-type variables but different indicators

Figure 11 shows another case that cannot be represented by one *C*-type variable. According to Definition 2, the corresponding DUCG with *C*-type variables should be as shown in Fig. 12. It is easy to prove that Figs. 11 and 12 are equivalent to each other in inference.

### Normalizing paths

**Fig. 13:**
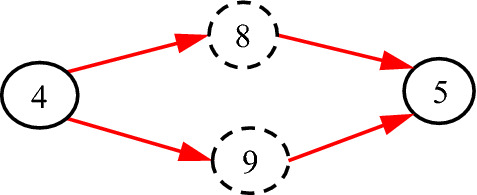
Illustration for repeated paths of *C*-type variables

**Fig. 14:**
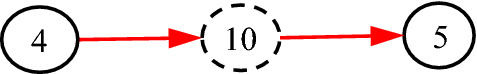
Normalized *C*-type path corresponding to Fig. 13

In practice, the repeated paths shown in Fig. 13 are possible. These repeated paths can be merged, that is, Fig. 13 can be calculated according to Fig. 14.

Figure 14 merges *C*_8_ and *C*_9_ in Fig. 13 into *C*_10_. The calculation of the merged parameters is as follows:

First, *I*_8;4_ and *I*_9;4_ in Fig. 13 are merged as *I*_10;4_ in Fig. 14. Next, *F*_5;10_ in Fig. 14 is equal to the sum of *F*_5;8_ = (*r*_5;8_/*r*_5_)*A*_5;8_ and *F*_5;9_ = (*r*_5;9_/*r*_5_)*A*_5;9_ in Fig. 13 as shown in Eq. (11).

11 $$\begin{gathered} F_{5;10} = F_{5;8} + F_{5;9} \hfill \\ f_{5;10} = f_{5;8} + f_{5;9} \hfill \\ \end{gathered}$$

#### Theorem 3

*Once a group of C-type variables share a same child variable and a same parent variable, this group of C-type variables can be merged as a single C-type variable along with its single I matrix. The merged F-type variable as the only output of the merged C-type variable is the sum of the group of F-type variables as the outputs of the group of C-type variables.*

#### Proof

Suppose the group of *C*-type variables are *C*_*i*_, *i* ∈ *S*_*C*_. They share a child variable *X*_*n*_ and a parent variable *V*_*m*_. Let *C*_*j*_ be the merged *C*-type variable, *j* ∉ *S*_*C*_, and *F*_*n;j*_ be the merged *F*-type variable that is the single output directed arc of the merged *C*-type variable. According to Eq. (1) and based on the original group of *C*-type variables, we have.

12 $$X_{n} = \sum\limits_{{i \in S_{C} }} {F_{n;i} C_{i} } = \sum\limits_{{i \in S_{C} }} {F_{n;i} I_{i;m} V_{m} } = \left( {\sum\limits_{{i \in S_{C} }} {F_{n;i} } } \right)V_{m}$$

Also, according to Eq. (1) but based on the merged *C*-type variable, we have

13 $$X_{n} = F_{n;j} C_{j} = F_{n;j} I_{j;m} V_{m} = F_{n;j} V_{m}$$

Let Eq. (12) equal to Eq. (13), we have

14 $$F_{n;j} = \sum\limits_{{i \in S_{C} }} {F_{n;i} }$$

Of course, the merged DUCG with *C*-type variable can be replaced in inference by the one without *C*-type variable.

## The Third-Party Verifications

To verify the diagnostic precisions and generalization ability of DUCG, we constructed six DUCG knowledge bases according to six chief complaints respectively, in which the *C*-type variables were used.

### Construction of DUCG with *C*-type variables

The construction steps are as follows.

***Step 1*** Determine the diseases that may cause the chief complaints across hospital departments, which means that the diseases are not limited in a specific hospital department and the triage may not be necessary, although the DUCG triage methodology has been presented in Bu et al. (2020).

**Fig. 15:**
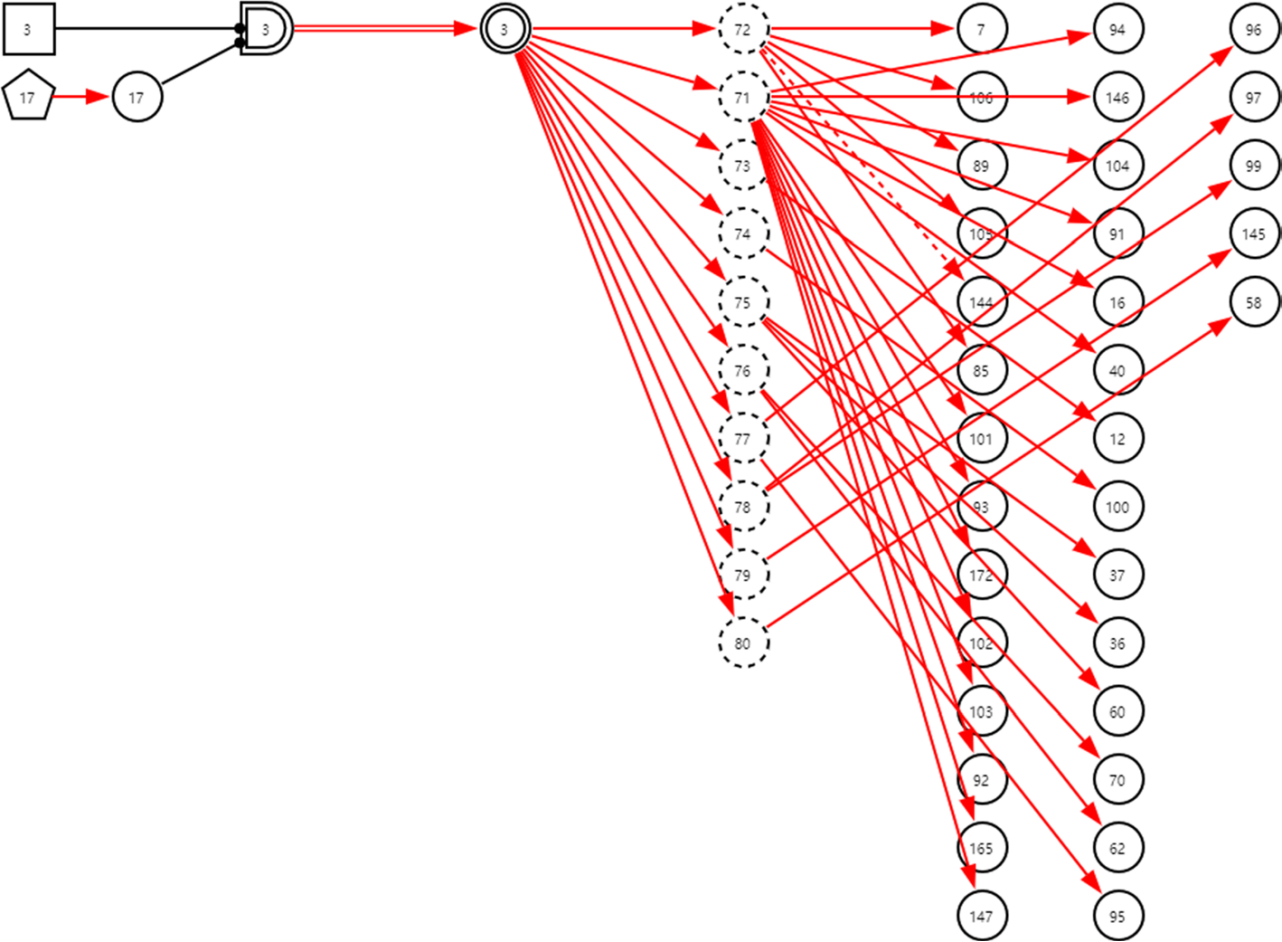
The subgraph with *C*-type variables for lyme disease under chief complaint arthralgia

**Fig. 16:**
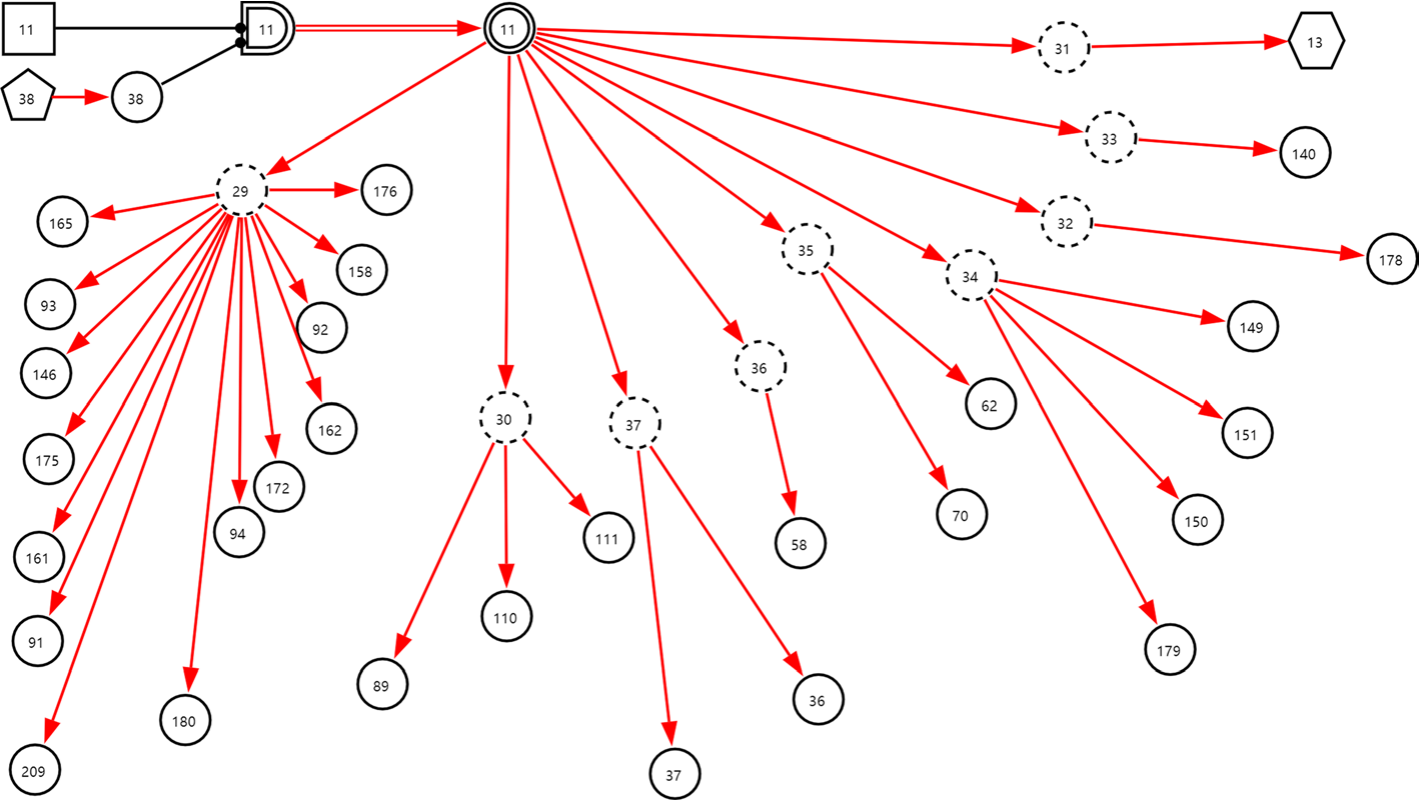
The subgraph with *C*-type variables for polymyositis under chief complaint arthralgia

***Step 2*** Construct the subgraph for every disease determined in step 1 as illustrated in Figs. 15 and 16 in which the symbols are described in Table 2. In subgraphs, the interpretability of DUCG knowledge bases is well demonstrated.

**Table 2:** Descriptions of the symbols in Figs. 15 and 16

Symbol	Variable	*n*	Description
and	*B*_*n*_ and *BX*_*n*_	3	Lyme disease
		11	Polymyositis
	*X*_*n*_	7	Erythema migrans
		12	ECG shows cardiac block
		16	Radiculopathy
		17	Experience of field travelling
		36	ESR
		37	CRP
		38	Sex
		40	Conjunctivitis ANA
		58	
		60	RF
		62	WBC
		70	HGB
		85	Skin rash
		89	Splenomegaly
		91	Arthralgia (acute or chronic)
		92	Arthralgia (large or small joint)
		93	Arthralgia (axis or peripheral)
		94	Arthralgia (self-limited or aggravating)
		95	CSF-WBC
		96	CSF-P
		99	CSF-PRO
		100	Abnormal ultrasonocardiography
		101	Headache
		102	Nausea
		103	Vomit
		104	Mental disorders
		105	Facial palsy
		106	Meningeal irritation sign
		110	Lymphadenectasis
		111	Hepatomegaly
		140	Chest CT shows interstitial pneumonia
		144	Testis swelling
		145	Borrelia burgdorferi-IgG
		146	Fever
		147	Cerebellar ataxia
		149	AST or ALT
		150	TBIL
		151	DBIL
		158	Myalgia
		161	Dysphagia
		162	Myasthenia
		165	Facet joint of hand pathological change
		172	Arthralgia (quantity)
		175	Limbs proximal myasthenia
		176	Weight loss
		178	Electromyogram shows myogenic muscular atrophy
		179	CK
		180	Dyspnea
		209	Anorexia
	*C*_*n*_	29	Symptom
		30	Sign
		31	Brucella culture
		32	Other imaging tests
		33	CT
		34	Blood biochemical test
		35	Anti-MCV antibody
		71	PLT
		72	CT shows sacroiliac joint injury
		73	ECG
		74	Ultrasonocardiography
		75	Rheumatic test
		76	Blood RT
		77	CSF RT
		78	CSF biochemical test
		79	Virus and infection related test
		80	Autoimmune antibody test
	*SX*_*n*_	13	Muscle biopsy shows myositis

**Fig. 17:**
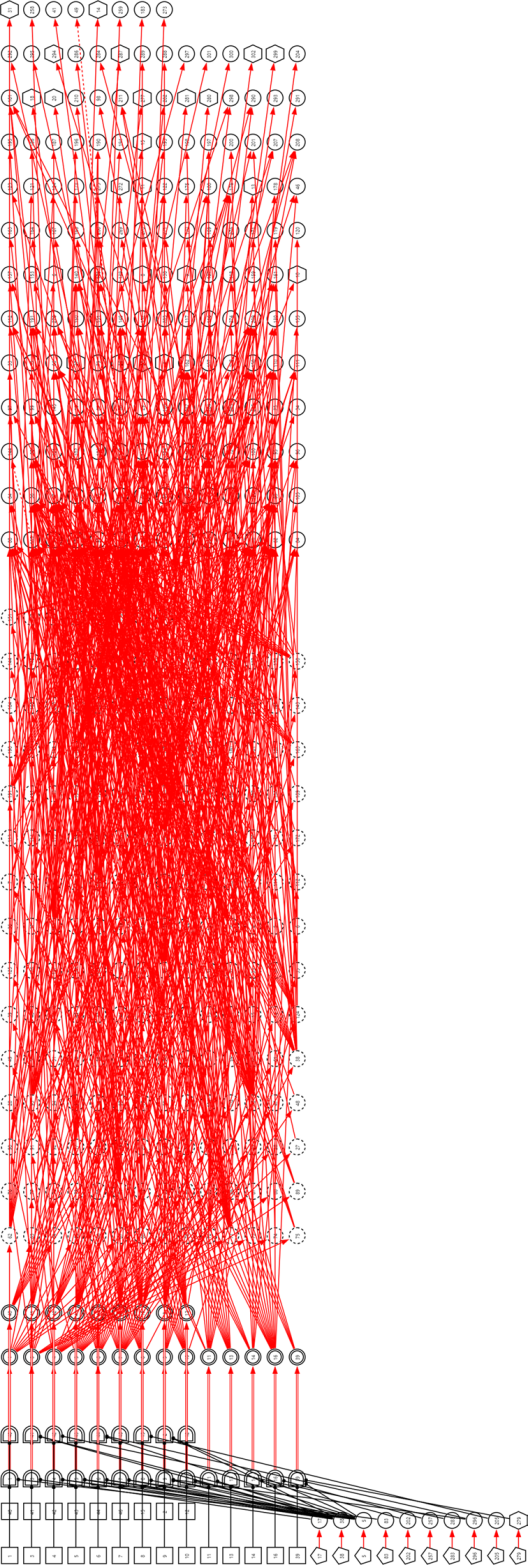
The DUCG including 23 diseases that may cause arthralgia

***Step 3*** Synthesize the subgraphs under a same chief complaint as a DUCG by fusing the same variables in different subgraphs. For example, the synthesized arthralgia DUCG is as shown in Fig. 17.

### Verifications, precisions and comparisons

After the DUCG construction, we tested its correctness carefully by using the case records in the hospital information system (HIS) of the knowledge base constructor’s hospitals as illustrated in Ref. (Zhang et al. 2021). Then, two groups of third-party verifications for six DUCG knowledge bases were performed independently to verify the generalization ability and diagnostic precisions of DUCG. The verifications done by Group 1 contain more diseases than Group 2, because Group 2 did verifications earlier than Group 1 when less diseases were considered. However, the diseases in Group 2 are all included in Group 1, so that we can compare the results of them in a comparable scale. The verifications were performed as follows:

1. Under each chief complaint, search the cases recorded in the HISs of the third-party hospitals for each disease.
2. For the total cases searched for each disease, randomly select no more than 10 cases for test.
3. Check the selected case record to ensure that it is in high quality, otherwise give up the case and make a new selection.
4. Manually input the evidences found in the tested case record into the DUCG cloud platform developed to implement the DUCG methodology.
5. Click the DUCG diagnosis function on the platform to find the possible diseases and rank them according to their conditional probabilities.
6. Compare the diagnosed diseases with the tested case record. If the diagnosed diseases with significant conditional probabilities cover the diseases in the record, and the clinical experts confirm that the diseases not in the record (if any) are also reasonable, label this tested case as “correct,” otherwise label it as “incorrect.” In fact, because of the uncertain quality, norm and format in the records, it was not easy to judge the correctness. In the confusing cases, discussions with clinical experts were the final means to make judgements.
7. Calculate the precision for each disease by the correct case number divided by the total tested case number of the disease.
8. Calculate the total precision for the DUCG of the chief complaint by the total correct case number divided by the total tested case number under the chief complaint.

As an example, the arthralgia DUCG verified in Group 1 is as shown in Fig. 17. Total 23 diseases are listed in Table 3, in which the 16 diseases in Group 2 are included. The verification results are shown in Tables 4, 5. The results for the other five chief complaints are in Tables 6, 7, 8, 9, 10 respectively in the Appendix. The total precisions from the two groups are listed and compared in Table 5. Note that the precisions from Group 2 are all 100%.

**Table 3:** The 23 diseases that may cause arthralgia, in which the diseases with “*” are not included in Group 2

Variable index	Disease	Abbreviate
1	Pseudogout	
2	Reactive arthritis	
3	Lyme disease	
4	Rheumatoid arthritis	RA
5	gout	
6	Adult still's disease	AOSD
7	Systemic lupus erythematosus	SLE
8	Sjögren's syndrome	SS
9	Osteoarthritis	OA
10	Ankylosing spondylitis	AS
11	Polymyositis	
12	Infectious arthritis	
13	Systemic sclerosis	SSc
14	Psoriatic arthritis	PsA
15	Brucellosis	
16	Tuberculosis	TB
39	Trauma*	
40	Relapsing polychondritis*	RPC
41	Polymyalgia arteritica*	PMR
42	Vasculitis*	
43	Sarcoidosis*	
44	Sports injury*	
46	Rheumatic fever*

**Table 4:** The precisions of the third-party verifications for arthralgia, in which the diseases with “*” are not included in Group 2

Disease	Total number of cases in Group 1; Group 2	Randomly selected and tested cases in Group 1; Group 2	Correct diagnoses in Group 1; Group 2	Precision in Group 1; Group 2 (%)
Gout	1129; 1733	10; 10	10; 10	100; 100
SLE	808; 1861	10; 10	10; 10	100; 100
PsA	14; 488	10; 10	10; 10	100; 100
Polymyositis	5; 184	5; 10	5; 10	100; 100
Sjögren's syndrome	95; 452	10; 10	10; 10	100; 100
Osteoarthritis	1388; 2586	10; 10	10; 10	100; 100
RA	2282; 3999	10; 10	10; 10	100; 100
Reactive arthritis	30; 76	10; 10	10; 10	100; 100
TB	67; 2074	10; 10	9; 10	90; 100
AS	44; 339	10; 10	10; 10	100; 100
AOSD	4; 80	4; 10	4; 10	100; 100
Infectious arthritis	5; 54	5; 10	5; 10	100; 100
SSc	9; 161	9; 10	9; 10	100; 100
Pseudogout	0; 2	0; 2	; 2	; 100
Brucellosis	1; 0	1; 0	1;	100;
Lyme disease	0; 0	0; 0	;	;
Sub-total	5881; 14,089	114; 132	113; 132	99.12; 100
Trauma*	876;	10;	10;	100;
RPC*	0;	0;	;	;
PMR*	0;	0;	;	;
Vasculitis*	0;	0;	;	;
Sarcoidosis*	0;	0;	;	;
Sports injury*	5;	5;	5;	100;
Rheumatic fever*	4;	4;	4;	100;
Total	6766;14,089	133;132	132;132	99.25; 100

SLE: systemic lupus erythematosus; RA: Rheumatoid arthritis; TB: Tuberculosis; PsA: psoriatic arthritis; AS: ankylosing spondylitis; AOSD: Adult Still's disease; SSc: systemic sclerosis

**Table 5:** The precisions of the third-party verifications for the six chief complaints, wherein the diseases in Group 2 are covered in Group 1

Chief complaint	Number of diseases in Group 1; Group 2	Number of total cases recorded in Group 1; Group 2	Randomly tested cases in Group 1; Group 2	Total precision in Group 1; Group 2 (%)	The lowest precision of a disease in Group 1; Group 2 (%)
arthralgia	23; 16	6766; 14,089	133; 132	99.25; 100	90; 100
dyspynea	28; 28	25,959; 65,834	202; 216	96.53; 100	80; 100
cough and expectoration	32; 28	102,935; 62,250	220; 223	99.55; 100	90; 100
epistaxis	24; 19	2033; 5913	137; 131	97.81; 100	90; 100
fever with rash	59; 17	13,290; 7935	386; 94	99.48; 100	90; 100
abdominal pain	99; 44	29,085; 35,631	612; 383	98.37; 100	83; 100

It is seen that the total precisions of the six DUCGs from the two groups respectively are very close to each other and no less than 96.5%, in which the lowest precision for all diseases was no less than 80%. The precision difference of the two groups is no more than |96.53 − 100|% = 3.47%. The mean precision difference of the six chief complaints is:

$$\frac{{\left| {99.12 - 100} \right| + \left| {96.53 - 100} \right| + \left| {99.49 - 100} \right| + \left| {99.14 - 100} \right| + \left| {100 - 100} \right| + |98.27 - 100|}}{6} = 1.24\%$$

### Verification discussions

For some relatively rare diseases, the case records were less than 10. In such cases, all the qualified records were selected. If there was no case found, the precision of this disease could not be calculated and was not considered in the precision calculations.

We believe that it is enough to test no more than 10 randomly selected cases for a disease in verifications, because 10 cases can cover most knowledge points related to the disease. If the knowledge base is correct, the test results will be correct, regardless of how many cases are tested. Given the total number of cases, if we increase the tested cases for every disease, only the tested cases of common diseases will be increased and the results will likely be correct, while the tested cases of rare diseases will not be increased due to the lack of cases, leading to an improper higher precision in total. The scientific way to perform the verification is to have the numbers of tested cases as equal as possible for all diseases. As a balance, we chosen to have no more than 10 tested cases.

The so-called “rare” disease means that it is rare under the chief complaint. A disease is rare under a chief complaint does not mean that it is also rare under other chief complaints.

It is easy to understand that only the discharged patient case records meet the high-quality requirement (the recorded information was sufficient and diagnosis was correct) for the third-party verifications. We did not use the outpatient case records for verifications, because it was hard to judge whether the outpatient diagnoses were correct or not. In general, the case record for a discharged patient contains more medical information than the case record of an outpatient. How to verify the diagnostic precision of DUCG conditional on less information for an outpatient is another issue and will be addressed elsewhere.

## Summery and discussions

The *C*-type variables are used only in the DUCG construction. Without *C*-type variables, the DUCG knowledge base is hard to be well organized and interpreted, and mistakes occur easily. The inference is based on the DUCG without *C*-type variables, which is automatically generated from the DUCG with *C*-type variables and is invisible.

Two groups of independent verifications for the six DUCG knowledge bases corresponding to six chief complaints verify that DUCG has strong generalization ability, which means that DUCG can be applied in any real application scenarios with almost the same precisions. This is because of the knowledge invariance.

The diagnostic interpretability of DUCG is provided by the generated sub-DUCG for each possible disease. A sub-DUCG is for a possible disease, in which all the evidences and causalities including the connected state-known variables and the isolated state-abnormal variables to this possible disease are displayed to the users in a graphical manner with text. More details can be found in Zhang et al. (2021).

DUCG does not deal with AI-aided medical image examination and medical sound recognition. They could be done by ML models. Hence, the relationship between DUCG and ML is cooperation.

In real applications, the AI-aided system should be able to recommend next medical checks based on the known information to collect further information for more accurate diagnosis. This will be discussed in another paper.

## Appendix

The diagnostic results of the five chief complaints (dyspnea, cough and expectoration, epistaxis, fever with rash, abdominal pain) are shown in following.

**Table 6:** Diagnostic precisions of DUCG for dyspnea, in which cases are randomly selected not more than 10 for each disease and “*” indicates only in Group 1

Disease	Total cases in Group 1; Group 2	Randomly selected and tested cases in Group 1; Group 2	Correct diagnoses in Group 1; Group 2	Precision in Group 1; Group 2 (%)
Carbon monoxide poisoning	58; 128	10; 10	10; 10	100; 100
Metabolic acidosis	9; 13	9; 10	9; 10	100; 100
HCM	10; 100	10; 10	9; 10	90; 100
Pulmonary infection	10; 18,421	10; 10	9; 10	90; 100
PAH	559; 70	10; 10	10; 10	100; 100
Interstitial lung disease	296; 0	10; 0	10;	100;
Pulmonary alveolar proteinosis	1; 2	1; 2	1; 2	100; 100
PE	101; 1080	10; 10	10; 10	100; 100
Heart failure	429; 2108	10; 10	9; 10	90; 100
HPS	0; 3	0; 3	; 3	; 100
DCM	151; 1330	10; 10	9; 10	90; 100
Anemia	3871; 3710	10; 10	10; 10	100; 100
Renal failure	1099; 2065	10; 10	10; 10	100; 100
Constrictive pericarditis	7; 67	7; 10	7; 10	100; 100
Pericardial effusion	300; 185	10; 10	10; 10	100; 100
Hemochromatosis	0; 1	0; 1	; 1	; 100
End-stage tumor	9; 220	9; 10	9; 10	100; 100
COPD	13,900; 12,872	10; 10	10; 10	100; 100
Laryngospasm	0; 0	0; 0	;	;
Foreign body in air passage	40; 146	10; 10	10; 10	100; 100
Obesity	5; 3	5; 3	5; 3	100; 100
Scoliosis	9; 306	9; 10	9; 10	100; 100
Pleural effusion	1469; 11,366	10; 10	10; 10	100; 100
Asthma	2294; 2409	10; 10	8; 10	80; 100
Bronchitis	1330; 9066	10; 10	9; 10	90; 100
Guillain–Barre syndrome	0; 7	0; 7	; 7	; 100
Myasthenia gravis	2; 156	2; 10	2; 10	100; 100
Psychology	0; 0	0; 0	;	;
Total	25,959; 65,834	202; 216	195; 216	96.53; 100

COPD: chronic obstructive pulmonary disease; HCM: hypertrophic cardiomyopathy; PAH: pulmonary artery hypertension; PE: pulmonary embolism; DCM: dilated cardiomyopathy; HPS: hepatopulmonary syndrome

**Table 7:** Diagnostic precisions of DUCG for cough and expectoration, in which cases are randomly selected not more than 10 for each disease and “*” indicates only in Group 1

Disease	Total cases in Group 1; Group 2	Randomly selected and tested cases in Group 1; Group 2	Correct diagnoses in Group 1; Group 2	Precision in Group 1; Group 2 (%)
Subacute thyroiditis	3; 33	3; 10	3; 10	100; 100
Pulmonary tuberculosis	1342; 1501	10; 10	10; 10	100; 100
Pneumothorax	951; 673	10; 10	10; 10	100; 100
Pulmonary abscess	453; 65	10; 10	10; 10	100; 100
Acute bronchitis	123; 1426	10; 10	10; 10	100; 100
Chronic bronchitis	2656; 5024	10; 10	10; 10	100; 100
Primary bronchogenic carcinoma	1023; 7430	10; 10	10; 10	100; 100
Bronchiectasis	6361; 2806	10; 10	10; 10	100; 100
COPD	129; 3245	10; 10	10; 10	100; 100
Pulmonary thromboembolism	18; 11	10; 10	10; 10	100; 100
Chronic pulmonary heart disease	6053; 2442	10; 10	10; 10	100; 100
Sarcoidosis	5; 675	5; 5	5; 5	100; 100
CVA	1; 276	1; 10	1; 10	100; 100
Upper respiratory tract infection	577; 4697	10; 10	10; 10	100; 100
Pneumonia	78,352; 16,721	10; 10	10; 10	100; 100
Heart failure	350; 6427	10; 10	10; 10	100; 100
Pleural effusion	37; 4428	10; 10	10; 10	100; 100
Bronchial asthma	4235; 2831	10; 10	9; 10	90; 100
Pericardial diseases	39; 50	10; 10	10; 10	100; 100
Vocal cord dysfunction syndrome	0; 286	0; 10	0; 10	0; 100
Nasal polyp	3; 1190	3; 10	3; 10	100; 100
IPF	49; 8	10; 8	10; 8	100; 100
Upper airway cough syndrome	0; 5	0; 5	0; 5	0; 100
Psychogenic cough	0; 0	0; 0	0; 0	0; 0
Tracheal collapse syndrome	0; 0	0; 0	0; 0	0; 0
EB	3; 0	3; 0	3; 0	100; 0
Reflux esophagitis	41; 0	10; 0	10; 0	100; 0
Diaphragmatic abnormalities	0; 0	0; 0	0; 0	0; 0
Sub-Total	102,804; 62,250	195; 223	194; 223	99.49; 100
Tracheobronchial foreign body*	109;	10;	10;	100;
Mediastinal lesions*	17;	10;	10;	100;
Diffuse interstitial lung disease*	2;	2;	2;	100;
Coronavirus disease 2019*	3;	3;	3;	100;
Total	102,935; 62,250	220; 223	219; 223	99.55; 100

COPD: chronic obstructive pulmonary disease; CVA: cough variant asthma; IPF: idiopathic pulmonary fibrosis; EB: eosinophilic bronchitis

**Table 8:** Diagnostic precisions of DUCG for epistaxis, in which “*” indicates only in Group 1

Disease	Total cases in Group 1; Group 2	Randomly selected and tested cases in Group 1; Group 2	Correct diagnoses in Group 1; Group 2	Precision in Group 1; Group 2 (%)
Malignant tumor of nasal cavity and paranasal sinus	7; 109	7; 10	7; 10	100; 100
Hemorrhagic nasal polyps	3; 94	3; 10	3; 10	100; 100
Nasal bone fracture	136; 26	10; 10	10; 10	100; 100
Fungal maxillary sinusitis	10; 26	10; 10	10; 10	100; 100
Acute leukemia	34; 631	10; 10	10; 10	100; 100
Inverting papilloma	15; 24	10; 10	10; 10	100; 100
Epistaxis	1089; 436	10; 10	10; 10	100; 100
Deviation of nasal septum	572; 870	10; 10	10; 10	100; 100
Nasal angioma	14; 80	10; 10	10; 10	100; 100
ITP	31; 562	10; 10	9; 10	90; 100
Maxillary sinus carcinoma	4; 138	4; 10	4; 10	100; 100
Nasopharyngeal carcinoma	85; 2906	10; 10	10; 10	100; 100
Ethmoid sinus fracture	0; 4	0; 4	; 4	; 100
Ethmoid sinus carcinoma	0; 3	0; 3	; 3	; 100
Atrophic rhinitis	8; 2	8; 2	8; 2	100; 100
HT	0; 2	0; 2	; 2	; 100
Foreign body in nasal cavity	3; 0	3; 0	3;	100;
Nasopharyngeal angiofibroma	1; 0	1; 0	1;	100;
Frontal sinus fracture	0; 0	0; 0	;	;
Sub-Total	2012; 5913	116; 131	115; 131	99.14; 100
AA*	8;	8;	8;	100;
MDS*	2;	2;	2;	100;
Hemophilia*	1;	1;	1;	100;
Hepatopathy*	10;	10;	8;	100;
Leptospirosis*	0;	0;	;	;
Total	2033; 5913	137; 131	134; 131	97.81; 100

ITP: Idiopathic thrombocytopenic purpura; HT: Hemorrhagic telangiectasia; AA: aplastic anemia; MDS: myelodysplastic syndrome

**Table 9:** Diagnostic precisions of DUCG for fever with rash, in which “*” indicates only in Group 1

Disease	Total cases in Group 1; Group 2	Randomly selected and tested cases in Group 1; Group 2	Correct diagnoses in Group 1; Group 2	Precision in Group 1; Group 2 (%)
Exanthema subitum	102; 44	10; 10	10; 10	100; 100
Hand foot mouth disease	200; 24	10; 10	10; 10	100; 100
Varicella	642; 93	10; 10	10; 10	100; 100
Infectious mononucleosis	8; 1019	8; 8	8; 8	100; 100
Herpes zoster	2348; 6652	10; 10	10; 10	100; 100
Measles	309; 69	10; 10	10; 10	100; 100
Dengue fever	2; 8	2; 8	2; 8	100; 100
Rubella	7; 7	7; 7	7; 7	100; 100
Herpetic angina	23; 6	10; 6	10; 6	100; 100
Scarlet fever	16; 6	10; 6	10; 6	100; 100
Typhoid fever	0; 6	0; 6	; 6	; 100
Tsutsugamushi disease	0; 1	0; 1	; 1	; 100
Hemorrhagic fever with renal syndrome	1; 0	1; 0	1;	100;
Epidemic cerebrospinal meningitis	0; 0	0; 0	;	;
Epidemic typhus	0; 0	0; 0	;	;
Endemic typhus	0; 0	0; 0	;	;
Paratyphoid fever	0; 0	0; 0	;	;
Sub-Total	3658; 7935	88; 94	88; 94	100; 100
HIV*	0;	0;	;	;
Systemic lupus erythematosus*	122;	10;	10;	100;
AOSD*	1;	1;	1;	100;
PM*	1;	1;	1;	100;
SS*	13;	10;	10;	100;
Erysipelas*	192;	10;	10;	100;
Rheumatic fever*	5;	5;	5;	100;
ANCA associated vasculitis*	0;	0;	;	;
Polyarteritis nodosa*	0;	0;	;	;
Nodular panniculitis*	8;	8;	8;	100;
APS*	3;	3;	3;	100;
Infective endocarditis*	1;	1;	1;	100;
Anthrax*	0;	0;	;	;
Contact dermatitis*	93;	10;	10;	100;
Melanoma*	91;	10;	10;	100;
Urticaria*	257;	10;	10;	100;
Drug induced rash*	142;	10;	10;	100;
Stevens Johnson syndrome*	0;	0;	;	;
Anaphylactoid purpura*	508;	10;	10;	100;
Vitiligo*	27;	10;	10;	100;
Scleroderma*	8;	8;	8;	100;
Furuncle*	380;	10;	10;	100;
Condyloma acuminatum*	13;	10;	10;	100;
Dermatophytosis*	124;	10;	10;	100;
Prurigo*	59;	10;	10;	100;
Syphilis*	9;	9;	9;	100;
Erythroderma*	36;	10;	10;	100;
Molluscum contagiosum*	17;	10;	10;	100;
Seborrheic dermatitis*	5919;	10;	10;	100;
Folliculitis*	35;	10;	9;	90;
Genital herpes*	6;	6;	6;	100;
Impetigo*	51;	10;	10;	100;
Eczema*	794;	10;	10;	100;
Cutaneous squamous cell carcinoma*	7;	7;	7;	100;
Psoriasis*	238;	10;	10;	100;
Wart*	288;	10;	9;	90;
Behcet syndrome*	7;	7;	7;	100;
Acne*	67;	10;	10;	100;
Amyloidosis cutis*	46;	10;	10;	100;
Typhus fever*	0;	0;	;	;
Carbuncle*	62;	10;	10;	100;
Cirrhosis*	2;	2;	2;	100;
Total	13,290; 7935	386; 94	384; 94	99.48; 100

*AOSD*: Adult still's disease; PM: Polymyositis; SS: Sjögren's syndrome; APS: antiphospholipid syndrome

**Table 10:** Diagnostic precisions of DUCG for abdominal pain, in which “*” indicates only in Group 1

Disease	Total cases in Group 1; Group 2	Randomly selected and tested cases in Group 1; Group 2	Correct diagnoses in Group 1; Group 2	Precision in Group 1; Group 2 (%)
Crohn's disease	8; 97	8; 10	7; 10	87.5; 100
Ulcerative colitis	125; 84	10; 10	9; 10	90; 100
Tuberculous peritonitis	10; 12	10; 10	9; 10	90; 100
Chronic pancreatitis	174; 12	10; 10	9; 10	90; 100
Acute pancreatitis	2124; 70 0	10; 10	10; 10	100; 100
Chronic cholecystitis	357; 615	10; 10	10; 10	100; 100
Acute cholecystitis	784; 342	10; 10	10; 10	100; 100
Anaphylactoid purpura	322; 461	10; 10	10; 10	100; 100
Ectopic pregnancy	461; 371	10; 10	10; 10	100; 100
Urinary calculi	2888; 503	10; 10	10; 10	100; 100
Angina pectoris	3; 522	3; 10	3; 10	100; 100
Renal failure	210; 897	10; 10	10; 10	100; 100
Chronic gastritis	3020; 1197	10; 10	10; 10	100; 100
Acute gastritis	457; 162	10; 10	10; 10	100; 100
Hepatitis	249; 410	10; 10	10; 10	100; 100
Saturnism	0; 10	0; 10	; 10	; 100
Gastric volvulus	2; 15	2; 10	2; 10	100; 100
Liver abscess	302; 74	10; 10	10; 10	100; 100
Gastrointestinal neurosis	137; 11	10; 10	9; 10	90; 100
Reflux esophagitis	933; 36	10; 10	10; 10	100; 100
Intestines and stomach cramps	2; 231	2; 10	2; 10	100; 100
Gastric or duodenal ulcer	255; 4893	10; 10	10; 10	100; 100
Pancreatic carcinoma	359; 85	10; 10	10; 10	100; 100
Herpes zoster	19; 232	10; 10	10; 10	100; 100
Liver carcinoma	443; 5900	10; 10	10; 10	100; 100
Hepatic rupture	49; 24	10; 10	10; 10	100; 100
Splenic rupture	311; 46	10; 10	10; 10	100; 100
Abdominal wall abscess	7; 21	7; 10	7; 10	100; 100
Hepatolithiasis	1242; 111	10; 10	10; 10	100; 100
Gastric carcinoma	297; 85	10; 10	10; 10	100; 100
Intraperitoneal tumor rupture	11; 12	10; 10	9; 10	90; 100
Miocardial infarction	65; 2185	10; 10	10; 10	100; 100
Gastrointestinal perforation	585; 121	10; 10	10; 10	100; 100
Appendicitis	1545; 10,368	10; 10	10; 10	100; 100
Enteritis	400; 4738	10; 10	10; 10	100; 100
Ischemicboweldisease	47; 25	4; 10	4; 10	100; 100
Diabetic ketoacidosis	102; 9	10; 9	10; 9	100; 100
Autoimmune pancreatitis	1; 6	1; 6	1; 6	100; 100
Eosinophilic gastroenteritis	5; 4	5; 4	5; 4	100; 100
Acute hemorrhagic necrotizing enteritis	0; 2	0; 2	; 2	; 100
Intestinal obstruction	2038; 1	10; 1	10; 1	100; 100
Porphyria	0; 1	0; 1	; 1	; 100
Pulmonary infection	1; 0	1; 0	1;	100;
Bile duct infection	3; 0	3; 0	3;	100;
Sub-Total	20,353; 35,631	346; 383	340; 383	98.27; 100
Fatty liver*	5107;	10;	10;	100;
Intussusception*	1091;	10;	10;	100;
Pelvic inflammatory disease*	818;	10;	9;	90;
Colorectal cancer*	503;	10;	10;	100;
Endometriosis*	234;	10;	9;	90;
Cirrhosis*	176;	10;	10;	100;
Carcinoma of gallbladder*	160;	10;	10;	100;
Cholangiocarcinoma*	126;	10;	10;	100;
Torsion of ovary and oviduct*	125;	10;	10;	100;
Oviduct ovarian abscess*	79;	10;	10;	100;
Acute cholecystitis with gangrene and perforation*	45;	10;	9;	100;
Dissection of aorta*	43;	10;	10;	100;
Acute pyelonephritis*	27;	10;	10;	100;
Systemic lupus erythematosus*	26;	10;	10;	100;
Renal infarction*	21;	10;	10;	100;
Small intestine tumor*	18;	10;	10;	100;
Portal vein thrombosis*	17;	10;	10;	100;
Splenic infarction*	17;	10;	10;	100;
Placental abruption*	17;	10;	10;	100;
Acute cystitis*	16;	10;	10;	100;
Acute urinary retention*	9;	9;	9;	100;
Intestinal tuberculosis*	6;	6;	5;	83;
Diverticular disease of colon*	6;	6;	6;	100;
Rupture of abdominal aortic aneurysm*	6;	6;	6;	100;
Spermatic cord torsion*	6;	6;	6;	100;
Dialysis related peritonitis*	4;	4;	4;	100;
Gallstone*	3;	3;	3;	100;
Pulmonary embolism*	3;	3;	3;	100;
Ovarian hyperstimulation syndrome*	3;	3;	3;	100;
Rupture of ovarian cyst*	3;	3;	3;	100;
Gastrointestinal neuroendocrineneoplasm*	3;	3;	3;	100;
Behcet's disease*	2;	2;	2;	100;
Splenic abscess*	2;	2;	2;	100;
Hepatic hydatid disease*	2;	2;	2;	100;
Chronic adrenocortical hypofunction*	2;	2;	2;	100;
Small intestinal diverticulum*	1;	1;	1;	100;
Acute adrenocortical hypofunction*	1;	1;	1;	100;
Antiphospholipid syndrome*	1;	1;	1;	100;
Costochondritis*	1;	1;	1;	100;
Gastrointestinal stromal tumor*	1;	1;	1;	100;
Spontaneous bacterial peritonitis*	1;	1;	1;	100;
Other abdominal tuberculosis*	0;	0;	;	;
Gastrointestinal diverticulum*	0;	0;	;	;
Esophageal diverticulum*	0;	0;	;	;
Takayasu arteritis*	0;	0;	;	;
Celiac artery compression syndrome*	0;	0;	;	;
Wilson's disease*	0;	0;	;	;
Gangrenous pyoderma*	0;	0;	;	;
Acute bacillary dysentery*	0;	0;	;	;
Polyarteritis nodosa*	0;	0;	;	;
Thallium poisoning*	0;	0;	;	;
Eosinophilic granulomatous polyangitis*	0;	0;	;	;
Rheumatoid vasculitis *	0;	0;	;	;
Lactose intolerance*	0;	0;	;	;
Pseudomembranous enteritis*	0;	0;	;	;
Total	29,085; 35,631	612; 383	602; 383	98.37; 100

## Footnotes

1 Corollary 15: $A_{{nk_{n} ;i}} V_{i} A_{{mk_{m} ;i}} V_{i} = \left( {A_{{nk_{n} ;i}} *A_{{mk_{m} ;i}} } \right)V_{i}$, in which $$\left( {A_{{nk_{n} ;i}} *A_{{mk_{m} ;i}} } \right) \equiv \left( {\begin{array}{*{20}c} {A_{{nk_{n} ;i1}} A_{{mk_{m} ;i1}} } & {A_{{nk_{n} ;i2}} A_{{mk_{m} ;i2}} } & \ldots & {A_{{nk_{n} ;ij}} A_{{mk_{m} ;ij}} } & \ldots & {A_{{nk_{n} ;iJ}} A_{{mk_{m} ;iJ}} } \\ \end{array} } \right)$$ Correspondingly, $$a_{{nk_{n} ;i}} *a_{{mk_{m} ;i}} \equiv \left( {\begin{array}{*{20}c} {a_{{nk_{n} ;i1}} a_{{mk_{m} ;i1}} } & {a_{{nk_{n} ;i2}} a_{{mk_{m} ;i2}} } & \ldots & {a_{{nk_{n} ;ij}} a_{{mk_{m} ;ij}} } & \ldots & {a_{{nk_{n} ;iJ}} a_{{mk_{m} ;iJ}} } \\ \end{array} } \right)$$ where, “*” is an AND/multiplication matrix operator specially defined in DUCG. In format, the * operator is similar to Hadamard product.
